# Subbing in: Associations Between Screen-Based Clinical Substitutions and Pediatric Clerkship Performance Outcomes

**DOI:** 10.1007/s40670-026-02651-5

**Published:** 2026-02-26

**Authors:** Alyssa L. MacMahon, Meaghan S. Wido, Steven J. Durning, Sami A. Abuhamdeh

**Affiliations:** 1 Department of Pediatrics, F. Edward Hébert School of Medicine, Uniformed Services University, Bethesda, MD USA; 2 Department of Health Professions Education, F. Edward Hébert School of Medicine, Uniformed Services University, Bethesda, MD USA

**Keywords:** Pediatrics clerkship, Clinical substitutions, Medical education, Performance outcomes

## Abstract

**Background:**

Clinical experiences foster medical students’ clinical reasoning and professional identity. When real patient encounters are not available, students often utilize screen-based simulation. While screen-based substitutions can be a comparable alternative (Meyer et al. Acad Psychiatry 47:181-186, 2023; Patel et al. Cureus 16:e54541, 2024), there is less clarity regarding frequency and type of experiences students substitute with simulation and how this relates to clinical competencies.

**Objective:**

This retrospective study seeks to quantify the trends of screen-based substitutions among pediatric clerkship students and investigate associations between substitutions and performance outcomes.

**Methods:**

Data were collected from the Uniformed Services University, Department of Pediatrics archival records (2018–2023) of students (*N* = 884) tracked clinical experiences, scores on the National Board of Medical Examiners ® (NBME) Pediatrics Subject exam, and a written History and Physical (H&P) exam. Analysis included descriptive statistics of clinical substitutions by type, location, and over time. Regression analysis compared the number of substitutions and performance outcomes.

**Results:**

Students substituted a mean of 0.79 (SD = 1.08) out of the 12 required clinical experiences. *Acute Gastroenteritis* was consistently and frequently substituted. Overall, substitutions increased significantly in 2020 compared to 2018 and 2019. There was a small but significant negative relationship between number of substitutions and NBME scores (*β* = -.06, *p* < .05), but no significant relationship with H&P scores (*β* = .02, *p* = .40).

**Conclusion:**

Increased use of substitutions since 2020 raises concerns about limitations in students' exposure to core clinical experiences. However, the minimal association between substitutions and performance outcomes suggests screen-based substitutions are still a reasonable alternative.

**Supplementary Information:**

The online version contains supplementary material available at 10.1007/s40670-026-02651-5.

## Introduction and Literature Review

Clinical experiences are highly valued during medical school because they not only expose students to a variety of patients and settings but also serve as the basis for specialty selection [3]. In line with Kolb’s experiential learning theory [4], in-person clinical experiences have traditionally been viewed as the optimal modality to teach, solidify and expand upon medical knowledge learned in the pre-clinical phase [5]. Specifically, an in-person encounter provides concrete experiences necessary for students to engage in reflective observation before moving toward abstract conceptualization and active experimentation in subsequent patient encounters. In acknowledgement of these benefits, ensuring medical students have in-person clinical experiences is also a requirement of the Liaison Committee on Medical Education and the Commission on Osteopathic College Accreditation. Nonetheless, because a full range of real patient encounters is not always available in the clinical setting, simulations are often offered as a substitute to real experiences.

High fidelity patient simulation, coupled with active learning, has the potential to engage learners in Kolb’s theory of experiential learning [6]. Screen-based simulation – a type of simulation where learning occurs via a digital interface on a computer or mobile device – is a frequently used modality in medical education. In general, screen-based simulations lead the learner through a patient encounter, prompting them to elicit a chief complaint, perform an exam, develop an assessment and differential diagnosis, and determine appropriate diagnostic testing and management. Substituting in-person clinical experiences with screen-based simulated patient encounters provides students with an opportunity to learn about important clinical concerns that they may not get to see in the clinic. Further, the screen-based format supports autonomy to complete a simulation at a time and place convenient for the student. The evolution of screen-based simulated patient care in medical education, particularly since the COVID-19 pandemic and the ongoing challenges of preceptor availability, has highlighted it as a compelling alternative to in-person patient care [1, 2]. For example, Myer and colleagues [1] found that overall Objective Structured Clinical Examination (OSCE) performance was non-inferior for clerkship students who participated in tele-simulation when compared to in-person learning. Further, Patel and colleagues [2] surveyed clerkship students following the pandemic and assessed the effectiveness of various learning strategies, finding that online simulation received the second highest rating. Nonetheless, if screen-based simulated patient encounters become the primary learning modality for core clinical experiences, it raises concerns about whether students are graduating with the necessary practical exposure to effectively manage common clinical scenarios. Many nursing students [7] and medical students [8, 9] feel that in-person patient care – compared to virtual encounters or screen-based simulation – better meets their needs for learning clinical skills and becoming acclimated to the clinical setting. On the other hand, research demonstrates that the use of screen-based simulation has varying association with performance outcomes: While nursing students had no significant differences in performance outcomes when comparing in-person pediatric clinical practice to virtual simulation training [10], medical students had “a small but positive relationship” between the number of in-person clinical encounters and clinical competence [11]. Given these inconsistent outcomes, further investigation is warranted to examine the associations between the use of screen-based simulation as a substitute for in-person patient care (henceforth referred to as “clinical substitutions”) and students’ pediatric clerkship performance outcomes.

As this study progressed, researchers found it necessary to take into consideration different types of performance outcomes and what they measure. For instance, assessments like the National Board of Medical Examiners ® (NBME) Subject exam are intended to measure medical students’ clinical knowledge and skills. Success on an exam like the NBME usually requires the student to take an “active role” in clinical learning experiences [12], which includes engaging with real patients in the clinical setting. The learning that takes place during a screen-based simulation has much more potential to be passive (eg, clicking through the encounter) and may hinder students’ development of these knowledge and skills. Other measures of performance, such as written work, may evaluate students' ability to synthesize information and articulate it clearly in a written format. In this instance, the use of clinical substitutions may or may not play a role in performance outcomes.

In addition to analysis of the associations between clinical substitutions and performance outcomes, understanding the trends of clinical substitutions may also help institutions to identify resource limitations at clerkship sites and make informed discussions about optimizing the learning environment across different sites. Despite the growing literature on screen-based simulation [13], the frequency and type of in-person clinical experiences substituted with screen-based simulation in pediatric clerkships remains understudied. While analyzing the rate and types of clinical substitutions, it is important to consider whether their use is influenced by external factors. For instance, during the COVID-19 pandemic, restrictions on medical student participation and hospital admissions may have impacted students’ opportunities to experience direct patient care, leaving clinical substitutions as one of the few options to gain experience. During this time, many institutions also shifted to virtual learning and/or shortened clerkship time for medical students, which may have further increased the use of clinical substitutions. Additionally, in the pediatric setting, the patient census predictably ebbs and flows [14], resulting in differences in availability of clinical encounters throughout the year. Another factor to consider is that not all clerkship sites have pediatric Graduate Medical Education (GME) programs, the absence of which may decrease the frequency of in-person subspeciality patient cases available for medical students to participate in. As we proceed through this project, our objective is to analyze the available data from multiple perspectives to identify associations between screen-based clinical substitutions in the pediatric clerkship, with performance outcomes, and with the above mentioned factors.

This study is guided by the following research questions:

1. What are the temporal trends of clinical substitutions in the pediatrics clerkship from 2018 to 2023?
2. Is there a statistically significant correlation between the number of reported clinical substitutions and performance outcomes among pediatric clerkship students?

## Methods

### Context

The Uniformed Services University (USU) is the United States’ only medical school dedicated specifically to the training of uniformed physicians. The four-year program has a 12-month traditional block clerkship that divides students’ time between six core clerkships and one selective clerkship. All students complete a pediatric clerkship rotation at one of 12 different military treatment facilities (MTFs), located on Army, Navy, and Air Force installations situated across the United States. During the pediatric clerkship, medical students are expected to observe and participate in care for patients in the general pediatrics clinic, inpatient pediatrics, and the newborn nursery. To ensure students are developing key pediatric clinical skills and knowledge, 12 required clinical experiences were specifically chosen by the pediatric clerkship director, in collaboration with USU faculty, as high yield experiences:

1. Health supervision visit for a 2–12 month old
2. Health supervision visit for a 15–60 month old
3. Adolescent History and Physical (H&P) (health supervision or acute visit)
4. Acute Otitis Media (AOM) or Middle Ear Effusion
5. Respiratory Disorder (asthma, upper respiratory infection, croup, pneumonia)
6. Fever (any cause)
7. Acute Gastroenteritis
8. Rash (any type)
9. Neonatal Hyperbilirubinemia
10. Chronic Medical Problems (cystic fibrosis, cerebral palsy, congenital heart disease, hematology/oncology, autism)
11. Disorder of growth (obesity, failure to thrive, short stature)
12. Attend Newborn Delivery

When real patient encounters are not available at the students’ respective MTFs, clinical substitutions are required with interactive virtual patient cases on Aquifer ® [15]. Each Aquifer ® case – which is peer-reviewed and evidence-based – prompts students to elicit a chief complaint, take a history, perform a physical exam, write a summary statement, formulate a differential diagnosis, perform diagnostic testing, and create an ongoing plan for management. Cases typically take students 40 min to complete. Students track their completion of the required clinical experiences or clinical substitutions, on a document called the “Clinical Tracker” that is submitted at the end of their pediatrics rotation. In this study, we used these Clinical Trackers to calculate the number of clinical experiences each student substituted. The required clinical experiences remained consistent throughout the study period.

To assess students’ performance outcomes within the pediatric clerkship, students take the NBME Pediatrics Subject exam, a standardized, timed (2 h and 45 min), 110 multiple-choice assessment. As part of the USU pediatrics clerkship, students must submit a written H&P exam that requires them to complete an in-depth analysis of a pediatric patient from the inpatient ward. The report is assessed by site directors (ie, designated, local faculty supervisor) using the validated Pediatric History and Physical Exam Evaluation (P-HAPEE) [16].

### Data Collection

Data were collected from the USU Department of Pediatrics electronic archival records (2018–2023) of students (*N* = 884) tracked clinical experiences, NBME Pediatrics Subject exam scores, and written H&P scores. The USU pediatrics clerkship director compiled the data into a centralized spreadsheet where names, corresponding clinical experience tracking, and assessment scores were recorded. All subsequent analyses and results are presented as aggregate data only, ensuring individual privacy. Ambiguous or missing records of Clinical Trackers and incomplete student exam records were excluded from analysis. This study was approved by the USU IRB, protocol number DBS.2020.076.

### Analysis

The overall trends in the number of clinical substitutions recorded on the Clinical Trackers were observed through the use of descriptive statistics.

To assess whether the number of clinical substitutions differed significantly during the COVID-19 pandemic, a multilevel negative binomial regression model, with a random intercept for site, was used. A multilevel approach was selected to account for the hierarchical structure of the data, with students nested within clerkship sites. A negative binomial distribution was used because the outcome (number of substitutions) was a count variable characterized by overdispersion (ie, the variance was significantly greater than the mean). Year was entered as a categorical variable, and the year 2020 – the year marking the onset of the COVID-19 pandemic – was designated as the reference category.

To investigate association with pediatric patient census throughout the year, we examined the number of clinical substitutions recorded per student by month. Secondary analyses used Tukey-adjusted pairwise comparisons to assess statistical significance between time of year students participated in the pediatric clerkship.

A secondary analysis used an independent samples t-test to investigate if there was a difference in mean number of clinical substitutions between pediatric clerkship sites with and without pediatric GME programs.

To examine the associations between number of clinical substitutions and performance outcomes, NBME scores were regressed on the number of substitutions using an ordinary least squared regression model with cluster-robust standard errors by clerkship site. Pre-clerkship NBME basic science exam scores (ie, exams taken during pre-clerkship) were also included as a covariate to control for them. The associations between number of clinical substitutions and H&P scores were examined using a multilevel model with a random intercept for clerkship site, again controlling for pre-clerkship NBME scores. Finally, both models were extended to test for the possibility of interactive effects (year X number of substitutions) on performance outcomes (both NBME and H&P scores). Model diagnostics indicated adequate linearity and no evidence of heteroscedasticity for either model.

## Results

### Trends in Clinical Substitutions

Descriptive analyses indicated that the 884 students substituted a mean of 0.79 experiences (SD = 1.08) out of the 12 required clinical experiences. The frequency distribution of clinical substitutions per student is shown in Table 1 and visualized in Supplemental Fig. 1.

**Table 1:** Frequency distribution of clinical substitutions per student

Total number of clinical substitutions	Number of students (Percent)
0	471 (53.28%)
1	231 (26.13%)
2	115 (13.01%)
3	42 (4.75%)
4	16 (1.81%)
5	6 (0.68%)
6	3 (0.34%)

^a^Percentages reflect the proportion of 884 students who reported each clinical substitution count

Table 2 displays the percentage of substitutions for each of the 12 required clinical experiences, calculated as the number of students who substituted a given experience divided by the total number of students who completed the experience as indicated by their Clinical Tracker. As can be seen, *Acute Gastroenteritis* followed by *Acute Otitis Media* accounted for a relatively high share of the substituted clinical experiences.

**Table 2:** Percentage of each of the 12 required clinical experiences substituted

Clinical experiences	Percent substituted	*n*
1. Health Supervision: 2–12 months	1.03%	9
2. Health Supervision: 15–60 months	1.73%	15
3. Adolescent H&P	3.35%	29
4. Acute Otitis Media	16.74%	145
5. Respiratory Disorder	2.66%	23
6. Fever	5.89%	51
7. Acute Gastroenteritis	27.22%	236
8. Rash	1.73%	15
9. Neonatal Hyperbilirubinemia	6.46%	56
10. Chronic Medical Problem	1.96%	17
11. Disorder of Growth	8.89%	77
12. Attended Newborn Delivery	2.99%	26

^a^Percentages reflect the proportion of 884 experiences (one per student) that were substituted for each condition

Table 3 shows the three most frequently substituted experiences for each of the six study years. As can be seen, *Acute Gastroenteritis* was the most frequently substituted experience for the extent of the study period, with *Acute Otitis Media* reaching similar frequency of substitution starting in 2020. Supplemental Fig. 2 visualizes the distribution of substitutions by experience over time (year).

**Table 3:** Most frequently substituted clinical experiences by year

Year	#1 substituted clinical experience (*n*, %)	#2 substituted clinical experience (*n*, %)	#3 substituted clinical experience (*n*, %)
2018	*Acute Gastroenteritis* (40, 25.3%)	*Disorder of Growth* (15, 9.49%)	*Neonatal Hyperbilirubinemia* (8, 5.06%)
2019	*Acute Gastroenteritis* (32, 21.8%)	*Disorder of Growth* (12, 8.16%)	*Neonatal Hyperbilirubinemia* (6, 4.08%)
2020	*Acute Gastroenteritis* (36, 29.3%)	*Acute Otitis Media* (27, 22%)	*Fever* (13, 10.6%)
2021	*Acute Gastroenteritis* (47, 27.8%)	*Acute Otitis Media* (47, 27.8%)	*Fever* (15, 8.88%)
2022	*Acute Gastroenteritis* (39, 27.5%)	*Acute Otitis Media* (33, 23.2%)	*Disorder of Growth* (16, 11.3%)
2023	*Acute Gastroenteritis* (42, 29.2%)	*Acute Otitis Media* (31, 21.5%)	*Disorder of Growth* (16, 11.15%)

**Fig. 1:**
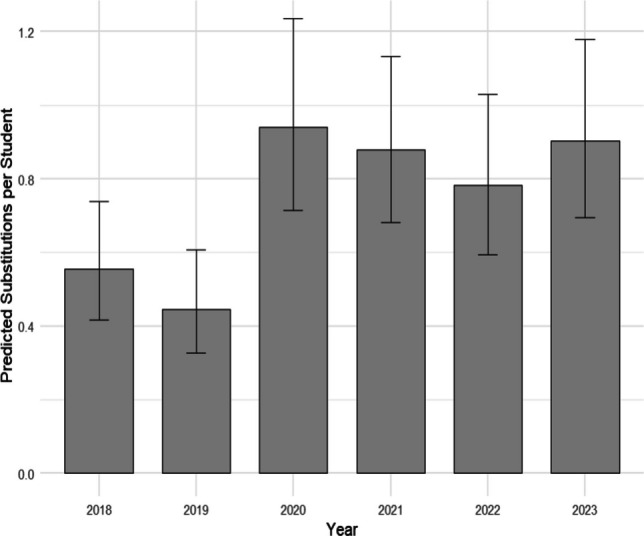
Mean number of clinical substitutions per student by year. ^a^Sample sizes by year: 2018 (*n* = 158), 2019 (*n* = 147), 2020 (*n* = 124), 2021 (*n* = 169), 2022 (*n* = 142), and 2023 (*n* = 144)

Results from a multilevel negative binomial model indicated that the number of clinical substitutions in 2020 increased significantly compared to the number of substitutions in both 2018 (Tukey-adjusted *p* = 0.014) and 2019 (Tukey-adjusted *p* < 0.001). The number of substitutions in 2020 did not, however, differ significantly from the number of substitutions in 2021, 2022, and 2023. Full model results are shown in Table 4, with mean number of clinical substitutions per student by year visualized in Fig. 1.

**Table 4:** Multilevel negative binomial model of clinical substitutions by year

Year comparison	Incidence rate ratio	95% CI lower	95% CI upper	*p* value
2018 vs 2020	0.590	0.430	0.810	0.001
2019 vs 2020	0.474	0.337	0.665	< 0.001
2021 vs 2020	0.935	0.701	1.246	0.647
2022 vs 2020	0.833	0.614	1.129	0.239
2023 vs 2020	0.962	0.713	1.298	0.798

^a^Random-intercept variance = 0.078 (SD = 0.279)

^b^Pairwise year contrasts were adjusted using Tukey’s method for multiple comparisons

^c^Zero-inflated multilevel negative binomial model did not improve fit compared with the standard multilevel negative binomial model reported here

Analysis of clinical experiences substitutions by time of year (see Table 5) found that the only statistically significant difference was between Block 6 (July–August time frame) and Block 7 (September–October time frame) (*p* = 0.0075).

**Table 5:** Percent of clinical experiences substituted by clerkship block

Block	Time of year	Number of students	Total clinical substitutions	Total possible experiences	Percent of clinical substitutions
1	January - February	130	117	1560	7.50%
2	February - March	132	116	1584	7.32%
3	March - April	51	48	612	7.84%
4	May - June	112	76	1344	5.65%
5	June - July	99	68	1188	5.72%
6	July- August	68	78	816	9.56%
7	September - October	120	65	1440	4.51%
8	October - November	126	98	1512	6.48%
9	November - December	46	33	552	5.98%

A subsequent analysis found that there was not a statistically significant difference in the mean number of clinical substitutions between pediatric clerkship sites that have pediatric GME (6 out of 12 sites) and sites without pediatric GME (*t* = −0.955, df = 399.84, *p* = 0.34).

### The Relationship Between Substitutions and Performance Outcomes

Descriptive statistics for NBME and H&P scores by year and substitution count are provided in Supplementary Table 1.

We assessed the association between clinical substitutions and pediatric clerkship performance outcomes with two measures: The NBME Pediatrics Subject scores and H&P scores. For NBME scores, results from the cluster-robust model indicated a very small but significant negative relationship between number of clinical substitutions and NBME scores (B = −0.48, *β* = −0.06, *p* < 0.05; see Table 6), indicating that for every one-unit increase in "number of clinical substitutions," the NBME score is predicted to decrease by 0.48 points, which is equivalent to approximately 6% of a standard deviation. A post-hoc analysis tested the possibility of non-linearity in the relationship between number of substitutions and NBME scores by comparing a linear-substitution model with a categorical (0/1/2 +) model; the categorical model did not improve fit. Adjacent contrasts (0 → 1 and 1 → 2 +) were also nonsignificant, providing no evidence of non-linearity.

**Table 6:** Ordinary least squares regression predicting NBME score by number of clinical substitutions

Predictor	B	SE	β	95% CI for B	*p* value
Number of clinical substitutions	−0.48	0.18	−0.06	−0.89 to −0.07	0.028
Pre-clerkship NBME	0.84	0.02	0.63	0.79 to 0.88	< 0.001

^a^Model R^2^ = 0.399; adjusted R^2^ = 0.397. Standard errors are clustered by pediatric clerkship site (12 sites)

For H&P scores, results from a multilevel model indicated no significant relationship between the number of substitutions and H&P scores (*B* = 0.07; *β* = 0.01, *p* = 0.72; see Table 7).

**Table 7:** Multilevel regression predicting H&P by number of clinical substitutions

Predictor	B	SE	β	95% CI for B	*p* value
Number of clinical substitutions	0.07	0.19	0.01	−0.31 to 0.45	0.72
Pre-clerkship NBME	0.19	0.03	0.18	0.12 to 0.25	< 0.001

^a^Random intercept variance (site) = 6.12 (SD = 2.47); residual variance = 36.43 (SD = 6.04)

The final set of analyses of interactive effects found no support for interactive effects. That is, there was no evidence that the relationship between number of clinical substitutions and students’ performance outcomes (ie, NBME and H&P scores) depended on year.

## Discussion

The objective of this study was to, first, to quantify the frequency, types, and temporal trends of clinical substitutions among pediatric clerkship students. Second, the study investigated the associations between clinical substitutions and students’ performance on the NBME and H&P.

### Overall Trends

From 2018 to 2023, over 90% of students had two or fewer clinical substitutions. Moreover, the frequency of substitutions did not vary by clerkship sites with or without pediatric GME programs. As medical schools aim to broaden their pediatric clerkship programs to include more diverse hospital locations, the USU pediatric clerkship provides an example. The results indicate that the USU pediatric clerkship can effectively deliver numerous, high-yield, real patient encounters at 12 varied sites, regardless of GME programs. When comparing across blocks (ie, time of year), findings showed only one significant difference in total number of clinical substitutions (Table 2). There are several anecdotal reasons for the increase in substitutions during July and August, such as decrease in pediatric illness while children are out of school or the start of the new GME year when Attendings’ focus is primarily on the incoming residents. Further investigation is needed to identify more definitive reasons for this increase.

### Frequency of Clinical Substitutions Over Time

Results showed a statistically significant increase in clinical substitutions from 2019 to 2020. This is in line with a substantial decrease in pediatric patient census during the COVID-19 pandemic [17, 18]. Additionally, at the USU, COVID-19 precautions in 2020 shortened the length of core clerkships (from five weeks to four), thereby reducing students’ overall time to care for patients with a variety of conditions. Furthermore, during 2020 and 2021, the students’ themselves may have been exposed to or contracted COVID-19, requiring transition to an isolated, virtual learning environment. Unexpectedly, the elevated numbers of clinical substitutions persisted throughout 2023, despite a learning environment that more closely resembled the pre-pandemic clerkships. For instance, there was a post-pandemic rise in pediatric patient census [19], a reduction in COVID severity and quarantine restrictions, as well as USU medical students returning to a regular clerkship schedule in 2022. One potential reason for the continued higher frequency of clinical substitutions through 2023 is learners’ attitudes toward online learning. Specifically, recent research found that many medical students in clerkships expressed a positive response to online learning and the autonomy it permitted within their schedules [20]. Consequently, the authors hypothesize that some medical students may have chosen to complete screen-based simulations at their convenience rather than waiting for a real-patient encounter.

### Most Frequently Substituted Clinical Experiences

The relatively high percentage of clinical substitutions for acute gastroenteritis may be surprising to most pediatricians considering this illness accounts “for 1.5 million office visits, 200,000 hospitalizations, and 300 deaths” of children in the United States each year [21]. And, while the rates of pediatric patients visiting both inpatient and outpatient for acute gastroenteritis decreased within the Military Health System during 2020 and 2021 [22], this does not explain the consistently high percentage of substitutions throughout the study period. The authors surmise that one potential reason for substitutions of these experiences may be related to misinterpretation of the clinical experience. For example, if medical students saw children with vomiting or diarrhea but failed to classify it as acute gastroenteritis, they may have felt the need to utilize the Aquifer case specifically labeled "acute gastroenteritis" to satisfy the requirement.

By 2020, the second most frequently substituted clinical experience became AOM. Although AOM has traditionally been one of the most common illnesses seen by pediatricians [23], recent research out of the United Kingdom has shown that from 2003–2019, occurrence of AOM declined by over 40% with the introduction of pneumococcal conjugate vaccines [24]. Additionally, researchers suspect reduced viral illness during the COVID period [25] may have impacted, and continues to impact, the number of visits for secondary infections like AOM. Consequently, pediatric clerkship students may not have had the opportunity to see children with AOM.

### Associations Between Clinical Substitutions and Performance Outcomes

The small, but significant negative correlation between clinical substitutions and NBME scores supports Kolb’s experiential learning theory that active clinical participation is crucial for knowledge and skill development associated with this assessment [4]. This paradigm is reflected in a study that found third year medical students’ OSCE scores were positively correlated to the total number of patients they cared for during their clerkship [11]. On the other hand, while screen-based simulations may help students to develop knowledge of systems (eg, immune, musculoskeletal, respiratory) as well as their application of concepts, diagnostic knowledge, interventions and management, there is less accountability for how actively engaged students are in learning. For instance, a student may "click through” a screen-based simulation without engaging with the material. Nevertheless, it must be emphasized that the magnitude of the substitution effect was extremely small – about 6% of one standard deviation – suggesting that, although in-person clinical encounters remain ideal, the modest decrement in NBME performance may be an acceptable tradeoff when clinical substitutions are needed to ensure coverage of required experiences.

The results of there being no significant relationship between clinical substitutions and performance on the H&P written exam are readily explicable. Namely, the open-resource nature of the retrospectively written H&P assignment measures students’ documentation, information literacy, and synthesis skills in an untimed setting, and it allows for review and refinement before submission. Consequently, in-person clinical experiences with patients likely do not contribute as much to students’ ability to perform on the written H&P. This is because the format of the H&P assignment effectively decouples raw data collection from the final presentation, allowing cognitive skills like organization and editing to dominate the score. This contrasts with the NBME, which assesses integrated clinical knowledge and reasoning in a timed setting without the use of external resources (eg, notes, textbooks), a format where the rich, integrated knowledge gained from varied, real-time patient encounters provides a critical and unique advantage for rapid pattern recognition and decision-making. All in all, the results of minimal association between clinical substitutions and performance outcomes suggests that the use of screen-based simulations is still a reasonable alternative to in-person patient encounters in a pediatrics clerkship.

### Limitations

There are limitations to this study. First, the data on the frequency and type of clinical substitutions were self-reported by the students, which may have led to an over- or under-estimation of the true use of clinical substitutions. The study team omitted all clinical trackers for which it was unclear if the student had done a clinical substitution or if it was incomplete. Second, due to changes in assignments and clinical performance grading policies within the USU Department of Pediatrics from 2018–2023, only two performance outcomes (ie, NBME and H&P scores) remained as valid and comparable for our analysis. Since 2018 the H&P has allowed students to resubmit the assignment for a potentially higher score, which may have inflated the means for the H&P. Lastly, given the Military Health System and the USU are nestled in a unique environment with a well-developed pediatrics clerkship curriculum, in addition to this study being limited to only the pediatrics clerkship, results may not be generalizable to a broad scope of institutions or other clerkships.

## Conclusion

In conclusion, the trends in clinical substitutions during the pediatric clerkship have revealed strengths as well as areas for improvement to ensure students fully engage in required clinical experiences during the pediatrics clerkship. The USU, overall, has a strong, structured clerkship curriculum with clear goals and objectives. The vast majority of pediatric clerkship experiences are conducted with in-person patient encounters that span a variety of inpatient and outpatient experiences and are coupled with immediate preceptor teaching and feedback. Within the pediatrics clerkship, students gain experience assessing acutely ill patients and screen-based simulation is used as an adjunct. In contrast to the USU pediatric clerkship, which is hosted at MTFs, some civilian medical schools' clerkships may be structurally more limited. This limitation often results in less exposure to inpatient and acutely ill patients, as well as preceptors' time with students which may be constrained by higher patient censuses. Factors such as these may result in clinical substitutions having a greater association with a decrease in performance outcomes, especially if used with higher frequency.

To address potential misinterpretations about the optional use of substitutions, the USU pediatric clerkship now provides clearer guidance during orientation, in the student handbook, and on Clinical Trackers. Site directors and preceptors received instruction reinforcing the need to ensure students engage in real patient encounters for high yield cases. Furthermore, we established a monitoring system of substitution rates across all sites to facilitate early intervention, including direct communication with site directors to address contributing factors (eg, lower newborn deliveries), as well as with students to re-emphasize the need to actively seek out experiences.

Additionally, while NBME scores were negatively correlated with clinical substitutions, it had a very small effect. This suggests that screen-based simulation with well-developed cases like those available on Aquifer ® [15] may not be immensely detrimental to students’ performance on assessments like the NBME and H&P during the pediatrics clerkship. We, nonetheless, recommend the continued prioritization of real patient encounters, specifically emphasizing the management of common pediatric illnesses, including AOM and gastroenteritis. Clinical substitutions may be optimally utilized for less common pediatric illnesses that students are unlikely to encounter during their clerkship but represent high-acuity or serious conditions that require reinforcement.

While this paper primarily examined the association of clinical substitutions and performance outcomes in a pediatrics clerkship, a compelling avenue for future research lies in understanding how these clinical substitutions could further serve as valuable complements to in-person learning regardless of specialty. For example, analyzing if completing both in-person clinical experiences and a complementary screen-based simulated encounter improves medical students’ clinical knowledge over just doing in-person.

Future research should also consider a more holistic approach that assesses the perspective of key players, such as preceptors and students, on how clinical experiences and clinical substitutions impact knowledge acquisition and performance outcomes. Furthermore, it would be beneficial to explore potential obstacles hindering student participation in direct patient care during their clerkship (eg, patient volume, preceptor accessibility) coupled with analysis of student engagement with screen-based simulation (eg, time spent, knowledge acquired). These data, in combination with an examination of detailed NBME score reports, may reveal correlations between specific clinical experiences replaced by screen-based simulation and performance in particular subject areas. Finally, additional projects should consider analyzing current trends and broadening investigation into multiple institutions and specialties.

## Supplementary Information

Below is the link to the electronic supplementary material.

Supplementary file1 (DOCX 142 KB)

## Data Availability

The raw data cannot be shared, but aggregated results are available upon request.

## References

[1] Meyer EG, Cozza KL, West JC, Hamaoka D. The effectiveness of online experiential learning in a psychiatry clerkship. Acad Psychiatry. 2023;47(2):181–6. 10.1007/s40596-023-01755-z. 36808570 10.1007/s40596-023-01755-z PMC9937738

[2] Patel R, Bannister SL, Degelman E, et al. Online learning in medical student clerkship: a survey of student perceptions and future directions. Cureus. 2024;16(2):e54541. 10.7759/cureus.54541[publishedOnlineFirst:20240220]. 38516469 10.7759/cureus.54541 PMC10956628

[3] Sathishkumar S, Thomas N, Tharion E, Neelakantan N, Vyas R. Attitude of medical students towards early clinical exposure in learning endocrine physiology. BMC Med Educ. 2007;7:30. 10.1186/1472-6920-7-30. 17784967 10.1186/1472-6920-7-30 PMC2045084

[4] Kolb DA. Experiential learning: experience as the scource of learning and development. Prentice Hall; 1984.

[5] Dornan T, Littlewood S, Margolis SA, Scherpbier A, Spencer J, Ypinazar V. How can experience in clinical and community settings contribute to early medical education? A beme systematic review. Med Teach. 2006;28(1):3–18. 10.1080/01421590500410971. 16627313 10.1080/01421590500410971

[6] Hogan M, Adebayo OB, Nela S, Silver S. High fidelity simulation in experiential learning. Secondary high fidelity simulation in experiential learning. 2020. https://www.saskoer.ca/instructionalstrategiesinhpe/chapter/high-fidelity-simulation-in-experiential-learning/. Accessed 2 June 2025.

[7] Roberts S, Warren T, Moore LC. COVID-19 pandemic: effects of replacing clinical hours with virtual simulation in BSN prelicensure nursing education. Nurs Educ Perspect. 2022;43(5):306–8. 10.1097/01.NEP.0000000000001002. 36037420 10.1097/01.NEP.0000000000001002

[8] Lashley PM, Sobers NP, Campbell MH, et al. Student satisfaction and self-efficacy in a novel online clinical clerkship curriculum delivered during the covid-19 pandemic. Adv Med Educ Pract. 2022;13:1029–38. 10.2147/AMEP.S374133. 36120394 10.2147/AMEP.S374133 PMC9473295

[9] Wenrich MD, Jackson MB, Wolfhagen I, Ramsey PG, Scherpbier AJ. What are the benefits of early patient contact? - a comparison of three preclinical patient contact settings. BMC Med Educ. 2013;13(1):80. 10.1186/1472-6920-13-80. 23731514 10.1186/1472-6920-13-80 PMC3674974

[10] Weston J, Zauche LH. Comparison of virtual simulation to clinical practice for prelicensure nursing students in pediatrics. Nurse Educ. 2021;46(5):E95-8. 10.1097/NNE.0000000000000946. 33186190 10.1097/NNE.0000000000000946 PMC8395966

[11] Kim JY, Myung SJ. Could clinical experience during clerkship enhance students’ clinical performance? BMC Med Educ. 2014;14(1):209–13. 10.1186/1472-6920-14-209. 25273978 10.1186/1472-6920-14-209 PMC4190391

[12] Ottenheijm RP, Zwietering PJ, Scherpbie AJ, Metsemakers JF. Early student-patient contacts in general practice: an approach based on educational principles. Med Teach. 2008;30(8):802–8. 10.1080/01421590802047265. 18608956 10.1080/01421590802047265

[13] Chernikova O, Heitzmann N, Stadler M, Holzberger D, Seidel T, Fischer F. Simulation-based learning in higher education: a meta-analysis. Rev Educ Res. 2020;90(4):499–541. 10.3102/0034654320933544.

[14] Capan M, Hoover S, Jackson E, Paul D, Locke R. Time series analysis for forecasting hospital census: application to the neonatal intensive care unit. Appl Clin Inform. 2016;7(2):275–89. 10.4338/ACI-2015-09-RA-0127. 27437040 10.4338/ACI-2015-09-RA-0127 PMC4941839

[15] Aquifer. Aquifer INC. 2025. https://aquifer.org/. Accessed 12 Nov 2024.

[16] King MA, Phillipi CA, Buchanan PM, Lewin LO. Self-directed rater training for pediatric history and physical exam evaluation (p-hapee) rubric, a validated written h&p assessment tool. MedEdPORTAL. 2017;13:10603. 10.15766/mep_2374-8265.10603. 30800805 10.15766/mep_2374-8265.10603 PMC6374741

[17] Fujiwara AS, Di Rocco JR, Hong TKF, Kimata C, Len KA. Impact of the COVID-19 pandemic on inpatient pediatric medical student education in Hawai’i. Hawaii J Health Soc Welf. 2024;83(7):192–9. 10.62547/FIMM9629. 38974805 10.62547/FIMM9629 PMC11224957

[18] Synhorst D, Bettenhausen J, Hall M, et al. Healthcare encounter and financial impact of COVID-19 on children’s hospitals. J Hosp Med. 2021;16(4):223–6. 10.12788/jhm.3572. 33734985 10.12788/jhm.3572 PMC8025590

[19] Children's Hospital Association. 4 trends in pediatric hospital utilization: a comparison of hospital utilization trends in acute care, surgery and emergency departments pre- and mid-pandemic. 2022. https://www.childrenshospitals.org/content/analytics/summary/4-trends-in-pediatric-hospital-utilization. Accessed 18 Mar 2025.

[20] Howell TC, Ladowski JM, Nash A, et al. The surgical clerkship in the COVID era: a natural language processing and thematic analysis. J Surg Res. 2024;299:155–62. 10.1016/j.jss.2024.04.016. 38759331 10.1016/j.jss.2024.04.016 PMC11189731

[21] Hartman S, Brown E, Loomis E, Russell HA. Gastroenteritis in children. Am Fam Physician. 2019;99(3):159–65. 30702253

[22] Lee EH, Solomon Z, Susi A, Chokshi, B, Hisle-Gorman, E, Gorman, G, & Nylund, C. Effects of the covid-19 period on pediatric inpatient and outpatient service utilization for gastroenteritis and urinary tract infection in US military health facilities. Poster Presentation at: The American Academy of Pediatrics National Conference & Exhibition; October 20-24, 2023; Washington, DC.

[23] Paul CR, Moreno MA. Acute otitis media. JAMA Pediatr. 2020;174(3):308. 10.1001/jamapediatrics.2019.5664. 31985755 10.1001/jamapediatrics.2019.5664

[24] Mohanty S, Podmore B, Cunado Moral A, et al. Incidence of acute otitis media from 2003 to 2019 in children </= 17 years in england. BMC Public Health. 2023;23(1):201. 10.1186/s12889-023-14982-8. (**[publishedOnlineFirst:20230130]**). 36717794 10.1186/s12889-023-14982-8 PMC9885604

[25] Taylor A, Whittaker E. The changing epidemiology of respiratory viruses in children during the COVID-19 pandemic: a canary in a COVID time. Pediatr Infect Dis J. 2022;41(2):e46-8. 10.1097/INF.0000000000003396. 35017455 10.1097/INF.0000000000003396 PMC8740030

