# Supplementary material for: Subbing in: Associations Between Screen-Based Clinical Substitutions and Pediatric Clerkship Performance Outcomes
Source: Med Sci Educ. 2026 Feb 26;36(2):829–37. doi: 10.1007/s40670-026-02651-5 (PMC13197555; doi:10.1007/s40670-026-02651-5)
Supplement: Supplementary file 1 — Supplementary file1 (DOCX 142 KB) [file 40670_2026_2651_MOESM1_ESM.docx]

SUPPLEMENTAL FIGURES AND TABLES

**Figure 1:** Distribution of Clinical Substitutions per Student

**
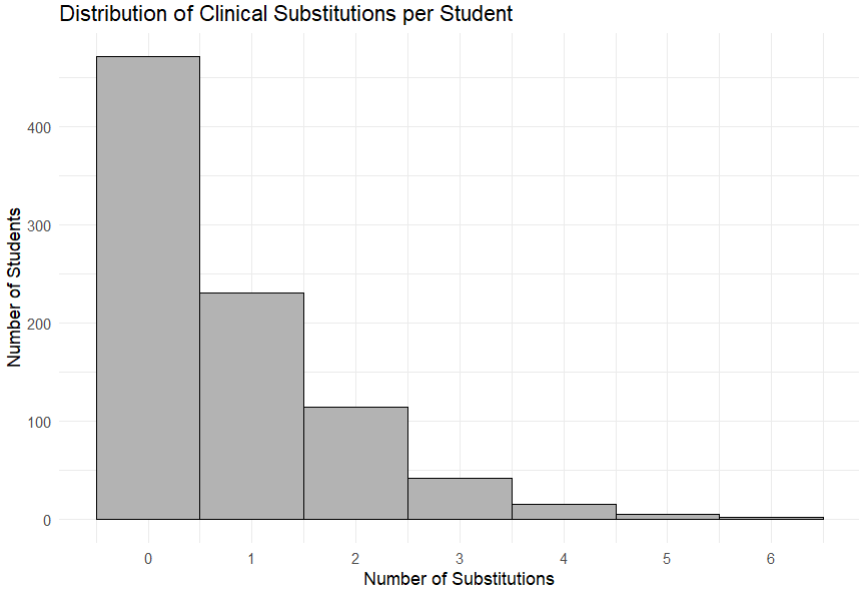
**

^a^ Total student sample size = 884.

**Figure 2:** Distribution of Clinical Substitutions by Experience Type and Year


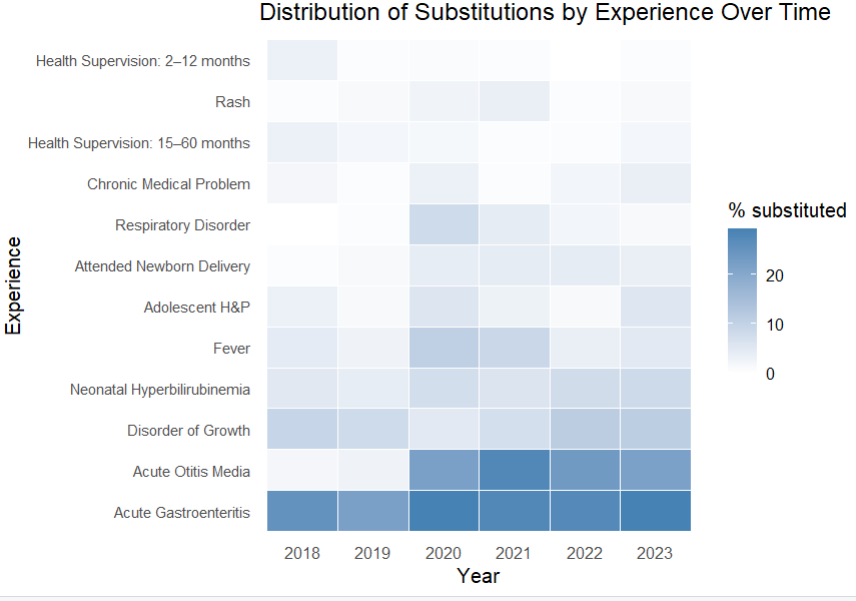


**Table 1:** NBME and H&P Descriptives by Substitution Count and Year

**2018 (N=158)**

| Substitutions | n | % of Students | NBME Mean (SD) | H&P Mean (SD) |
| --- | --- | --- | --- | --- |
| 0 | 98 | 62.0% | 74.13 (7.10) | 36.49 (6.73) |
| 1 | 40 | 25.3% | 73.12 (7.50) | 35.42 (7.86) |
| 2+ | 20 | 12.7% | 73.00 (8.19) | 35.35 (6.22) |

**2019 (N=147)**

| Substitutions | n | % of Students | NBME Mean (SD) | H&P Mean (SD) |
| --- | --- | --- | --- | --- |
| 0 | 95 | 64.6% | 75.62 (8.02) | 37.09 (6.02) |
| 1 | 40 | 27.2% | 71.50 (8.20) | 36.55 (7.87) |
| 2+ | 12 | 8.2% | 73.75 (8.74) | 38.08 (4.29) |

**2020 (N=124)**

| Substitutions | n | % of Students | NBME Mean (SD) | H&P Mean (SD) |
| --- | --- | --- | --- | --- |
| 0 | 60 | 48.4% | 70.22 (8.92) | 35.47 (6.57) |
| 1 | 27 | 21.8% | 70.48 (8.34) | 36.69 (6.10) |
| 2+ | 37 | 29.8% | 70.00 (6.92) | 39.67 (5.81) |

**2021 (N=169)**

| Substitutions | n | % of Students | NBME Mean (SD) | H&P Mean (SD) |
| --- | --- | --- | --- | --- |
| 0 | 78 | 46.2% | 73.12 (7.35) | 38.78 (6.74) |
| 1 | 47 | 27.8% | 73.09 (8.08) | 39.30 (6.79) |
| 2+ | 44 | 26.0% | 71.98 (7.77) | 39.98 (7.01) |

**2022 (N=142)**

| Substitutions | n | % of Students | NBME Mean (SD) | H&P Mean (SD) |
| --- | --- | --- | --- | --- |
| 0 | 68 | 47.9% | 73.87 (9.57) | 39.51 (6.61) |
| 1 | 42 | 29.6% | 73.21 (8.58) | 39.36 (5.59) |
| 2+ | 32 | 22.5% | 71.94 (11.04) | 39.44 (6.65) |

**2023 (N=144)**

| Substitutions | n | % of Students | NBME Mean (SD) | H&P Mean (SD) |
| --- | --- | --- | --- | --- |
| 0 | 72 | 50.0% | 71.88 (8.77) | 41.64 (5.03) |
| 1 | 35 | 24.3% | 75.09 (8.53) | 40.83 (4.68) |
| 2+ | 37 | 25.7% | 72.89 (6.65) | 39.54 (5.29) |
